# Differences in Proinflammatory Property of Six Subtypes of Peroxiredoxins and Anti-Inflammatory Effect of Ligustilide in Macrophages

**DOI:** 10.1371/journal.pone.0164586

**Published:** 2016-10-07

**Authors:** Li-Xue Zhao, Jun-Rong Du, Hong-Jing Zhou, Dong-Ling Liu, Man-Xia Gu, Fang-Yi Long

**Affiliations:** Department of Pharmacology, Key Laboratory of Drug Targeting and Drug Delivery Systems, West China School of Pharmacy, Sichuan University, Chengdu, China; Universitatsklinikum Freiburg, GERMANY

## Abstract

**Background:**

Peroxiredoxins (Prxs) are proposed to function as damage-associated molecular patterns (DAMPs) and contribute to post-ischemic neuroinflammation and brain injury by activating Toll-like receptor (TLR) 4 at the acute and subacute phases after ischemic stroke. However, there are few studies concerning the inflammatory profiles of six distinct subtypes of Prxs (Prx1–Prx6). Our previous study demonstrated that the protective effect of ligustilide (LIG) against cerebral ischemia was associated with inhibition of neuroinflammatory response and Prx/TLR4 signaling in rats. Herein, the present study explored the inflammatory members of Prxs and the effect of LIG on their inflammatory responses in macrophages.

**Methodology/Principal Findings:**

The murine RAW264.7 macrophages were treated with each of exogenous recombinant Prxs at a range of 1 to 50 nM for 24 h. The WST-1 test showed that Prx3 exhibited a significant cytotoxicity, whereas the rest five Prxs did not affect cellular viability. The quantitative measurements with spectrometry or ELISA indicated that three subtypes, Prx1, Prx2 and Prx4, increased production of proinflammatory mediators, including nitric oxide (NO) metabolites, tumor necrosis factor-α (TNF-α), and interleukin-6 (IL-6) in a concentration-dependent manner. Immunostaining demonstrated that 20 nM Prx1, Prx2 or Prx4 significantly increased expression of TLR4 and iNOS and nuclear translocation of NF-κB p65. However, Prx5 and Prx6 showed no poinflammatory effect in macrophages. Remarkably, LIG treatment effectively inhibited the inflammatory response induced by Prx1, Prx2 and Prx4.

**Conclusion:**

Three members of Prxs, Prx1, Prx2 and Prx4, are inflammatory DAMPs that induce TLR4 activation and inflammatory response in macrophages, which is effectively inhibited by LIG. These results suggest that inflammatory Prxs-activated macrophages may provide a novel cellular model for screening the potential inhibitors of DAMPs-associated inflammatory diseases such as stroke. Moreover, selective blocking strategies targeting the inflammatory subtypes of Prxs probably provide promising therapeutic approaches with a prolonged time window for stroke.

## Introduction

Neuroinflammation has come to denote central nervous system (CNS)-specific inflammatory responses that are implicated in the progression of cerebral ischemic injury [1]. It is well known that the major inflammatory cells in ischemic brain are the activated macrophages, consisting of resident microglia in brain and infiltrating monocytes from peripheral blood. A number of studies have demonstrated that overactivated macrophages in brain play a pivotal role in post-ischemic neuroinflammatory response by induction of various proinflammatory mediators production [1,2]. Consistent with this notion, various anti-inflammatory neuroprotectants, such as anti-intracellular cell adhesion molecule 1 (ICAM-1) antibody (Enlimomab) and cyclooxygenase inhibitor (Paracetemol), have been evaluated for their therapeutic potential in ischemic stroke [3]. To date, however, these attempts have not yielded the desired outcomes in clinical trials because of either disappointing efficacy or serious side-effects, and the optimal therapy for post-ischemic neuroinflammation and brain injury has not yet been developed. Therefore, there is a huge unmet need for the development of innovative therapeutic strategies targeting post-ischemic neuroinflammatory cascades in ischemic stroke.

Toll-like receptor 4 (TLR4) constitutively expressed in various brain cells and blood-borne immune cells has been demonstrated to be intimately associated with experimental and clinical neuroninflammatory responses and outcomes in ischemic brain [4,5]. Cerebral ischemia stress robustly induces the extracellular release of damage-associated molecular patterns (DAMPs) from necrotic and dying neural cells within ischemic territory, which may function as the endogenous ligands for TLR4 and induce the activation of TLR4/nuclear factor κB (NF-κB) signaling pathway, thereby enhancing both innate and adaptive immunity and promoting post-ischemic neuroinflammation and brain infarction [6,7]. Recent studies have revealed two major protein DAMPs with a differential temporal profile, high mobility group box-1 protein (HMGB-1) and peroxiredoxins (Prxs) [8,9]. In contrast to the early release of HMGB-1 within 6 h after brain ischemia, Prxs are extracellularly released from damaged brain cells predominantly 12–72 h after stroke onset in mice [9]. Remarkably, the extracellular Prxs without antioxidant capacity may act as the specific DAMPs responsible for TLR4 activation and promote post-ischemic neuroinflammation and neuronal damage via release of neurotoxic cytokines during the acute phase, and production of IL-23 and IL-12 during the delayed phase that thereby causes release of neurotoxic IL-17 and IFN-γ and late brain injury [9]. Moreover, systemic administration of a mixture of antibodies specific for Prxs even at 12 h after cerebral ischemia significantly ameliorated both cerebral infarct and motor deficits in mice [9]. It is reported that the mammalian Prxs family has six subtypes (Prx1–Prx6) with brain cell type-specific expression patterns: Prx1 and Prx6 are expressed in astrocytes and oligodendrocytes, while Prx2–Prx5 are mainly expressed in neurons [10–12]. To the best of our knowledge, the inflammatory profile of each subtype of Prxs has not been investigated in macrophages *in vitro*, which has become an obstacle to translational medicine research. Therefore, identifying the specific Prx(s) that activate TLR4 may promote the development of novel aseptic neuroinflammation models *in vitro* and innovative approaches with a prolonged time window for the treatment of neuroinflammation and brain injury after ischemic stroke.

In addition, Ligustilide (3-butylidene-4,5-dihydrophthalide, LIG, S1 Fig) is a main effective component of various traditional Chinese medicines, such as *Radix Angelicae sinensis* and *Ligusticum chuanxiong* [13]. We have reported the potent anti-neuroinflammatory and neuroprotective effects of LIG against various cerebral ischemic insults *in vivo*, and the strong inhibitory effect of LIG on inflammatory response induced by lipopolysaccharide (LPS), a natural ligand for TLR4, in primary rat cerebral microglia *in vitro* [14,15]. Moreover, our recent study showed that LIG protected against ischemic brain injury, accompanied by inhibition of extracellular release of Prx5 and Prx6, TLR4/NF-κB signaling and neuroinflammatory response [16]. Therefore, the present study explored the inflammatory members of Prxs and the effect of LIG on their inflammatory response in cultured murine RAW246.7 macrophages.

## Materials and Methods

### Reagents

Recombinant mouse Prxs were purchased commercially: Prx1 and Prx5 (Batch No: 50552-mM08E and 50551-M07E) from Sino Biological (Beijing, China), Prx2–Prx4 and Prx6 (Batch No: RPF757Mu01, RPF753Mu01, RPF754Mu01 and RPF756Mu01) from USCN Life Science (Wuhan, Hubei, China). The primary antibodies used in this study, including iNOS, TLR4 and NF-κB p65, were purchased from Boster Biological Technology (Wuhan, Hubei, China), Santa Cruz Biotechnology (Heidelberg, Germany), and Zhongshan Jinqiao Biology (Beijing, China), respectively. WST-1 Cell Proliferation and Cytotoxicity Assay Kit were purchased from Beyotime Biological Technology (Haimen, Jiangsu, China).

Prx1–Prx6 were prepared as a 10 μM stock solution in sterile water following the manufacturer's recommended procedure, while LIG (purity > 98.5%, S1 Fig), prepared by a well-established procedure in our laboratory (Kuang et al., 2008), was dissolved in DMSO as a 50 mM stock solution. Both Prx and LIG stock solutions were kept at -80°C, and diluted with cell culture medium before use. A final concentration of 0.1% DMSO was used as vehicle for the treatment of the control cultures.

### Cell culture and treatment

The murine macrophage-like cell line RAW264.7 was obtained from KeyGEN Biotechnology (NanJing, Jiangsu, China) and cultured in RPMI1640 medium (Gibco, Carlsbad, CA, USA) supplemented with 10% fetal bovine serum (FBS; Beijing Minhai Biotechnology, Beijing, China), penicillin (100 U/mL), streptomycin (100 mg/L), and L-glutamine (2 mM) in a humidified atmosphere of 95% air and 5% CO_2_ at 37°C.

On the day of the experiment, macrophage cells were seeded in 96-well plates and left to acclimate for 24 h. The culture medium was then replaced with fresh medium and the cells were pretreated with or without different concentrations of LIG for 1 h, followed by co-treatment with each Prx at the indicated concentration(s) for another 24 h. Cellular supernatants were harvested and stored at -80°C until further measurement of the levels of proinflammatory mediators, and the remaining cells were used for immunostaining.

### Cellular viability assay

The effects of distinct Prxs with or without LIG on the viability of RAW264.7 cells were assessed using the WST-1 assay as described previously [17]. In brief, after treatment with different Prx in the absence or presence of LIG at the indicated concentrations for 24 h, the cells in 96-well plate (1×10^5^ cells/well) were washed with serum-free medium and incubated with the WST-1 reagent (10 μL/well) in fresh serum-free medium at 37°C for an additional 2 h. At the end of the incubation period, the intensity of the color formation was detected using a microplate reader at 450 nm (Bio-Rad, Hercules, California, USA). Data are expressed as a percentage of control treatment alone [17].

### Nitrite assays

In order to detect the nitric oxide (NO) production in macrophages, 5 × 10^5^ cells per well were treated with Prx in the absence or presence of LIG at the indicated concentrations for 24 h, and then 100 μL supernatant of cells was harvested for determination of NO contents by measuring nitrite levels using NO assay kit (JianCheng Bioengineering Institute; NanJing, Jiangsu, China) according to the manufacturer's instruction [15].

### Measurement of cytokine levels

For the measurement of tumor necrosis factor α (TNF-α) and interleukin-6 (IL-6) production in macrophages, cells (5 × 10^5^ cells per well) were treated with Prx in the absence or presence of LIG at the indicated concentrations for 24 h. And then the culture supernatants were collected for cytokines measurement using ELISA kits (Dakewe Biological Technology; Shenzhen, Gungdong, China) according to the manufacturer's instruction [15].

### Immunocytochemistry and Immunofluorescence analysis

Immunocytochemistry analysis was used to detect the expression of iNOS and TLR4 in RAW264.7 cells according to the SABC kit procedure as reported previously [15]. In brief, the cells at a density of 1 × 10^4^ cells/mL were treated with Prx in the absence or presence of LIG at the indicated concentrations for 24 h. The cells were then fixed with 4% paraformaldehyde for 30 min at 37°C and washed with PBS for 3 times. The fixed cells were then permeabilized with 0.3% Triton X-100 in PBS for 30 min at 37°C and washed with PBS for 3 times. The permeabilized cells were then rinsed with 3% H_2_O_2_ for 30 min to block endogenous peroxide activity and incubated with 5% bovine serum albumin from the SABC kit for 1 h to block nonspecific binding. The cells were then incubated with rabbit polyclonal antibody against iNOS or TLR4 (1:100), respectively, overnight at 4°C, followed by detection with a biotinylated secondary antibody and the SABC kit at 37°C for 1 h, respectively. Immunoreactions were visualized using 3, 3’-diaminobenzidine tetrahydrochloride (DAB; Boster Biological Technology) as a chromagen. The expression of iNOS and TLR4 in cells was observed under microscope (Nikon, Japan), and photomicrographs were acquired using a 40 × objective and processed using SPOT advanced software 4.6 (SPOT Imaging Solutions, Sterling Heights, MI, USA) [15].

For examination of the nuclear translocation of NF-κB p65, the cells were incubated with rabbit polyclonal antibody against NF-κB p65 (1:100), followed by detection with a fluorescein conjugated secondary antibody (1:100; Boster Biological Technology) at 37°C for 1 h. After washing, the nuclei were counterstained with 2-(4-Amidinophenyl)-6- indolecarbamidine dihydrochloride (DAPI; Boster Biological Technology). The subcellular localization of NF-κB p65 was observed using a fluorescence microscope (Nikon), and photomicrographs were acquired as described above [18].

### Statistical analysis

Data are expressed as the mean ± SEM. of at least three independent experiments. To compare three or more groups, one-way analysis of variance was used, followed by a Newman-Keuls post-hoc test. Statistical analyses were performed with GraphPad Prism software, version 5.01 (GraphPad Software Inc., San Diego, CA, USA). Values of *P* < 0.05 were considered statistically significant.

## Results

### Effects of Prxs on macrophage cellular viability

**Fig 1:**
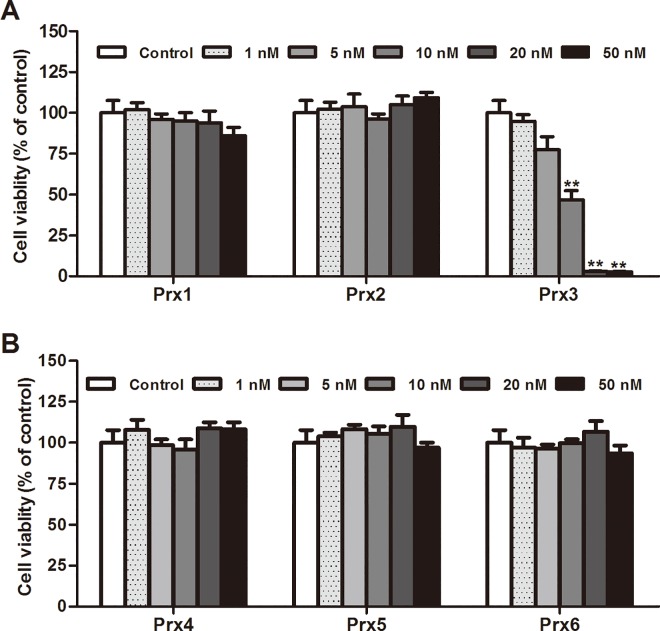
Effects of Prx1~Prd6 on cellular viability of murine RAW264.7 macrophages. RAW264.7 cells were treated with each Prxs at concentrations ranging from 1 to 50 nM for 24 h. The cell viability was examined by the WST-1 assay and the results are expressed as a percentage of control group. Data are representative of results obtained from three independent experiments with 6 replicates. **P < 0.01 versus control group.

The WST-1 assay was applied to determine the effect of each Prx on the cellular viability of murine RAW264.7 macrophages. As shown in Fig 1, the present results showed that 24 h treatment with Prx 1, 2, 4, 5 or 6 at concentrations ranging from 1 to 50 nM had no significant effect on RAW264.7 cell viability (*P* > 0.05), whereas Prx 3 showed a concentration-dependent cytotoxicity, leading to a significant decrease in cell viability at doses of 10 nM and greater levels (*P* < 0.01).

### NO production induced by Prxs in macrophages

**Fig 2:**
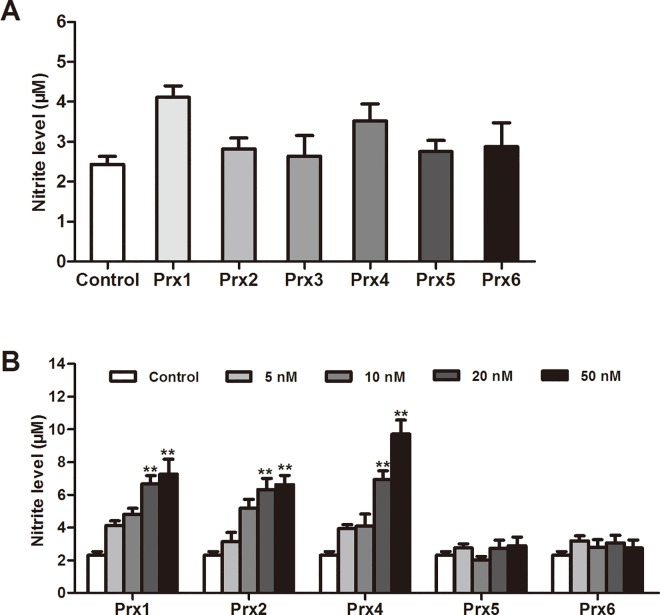
Effects of Prxs on NO production in murine RAW264.7 macrophages. RAW264.7 cells were treated with Prx at the indicated concentrations for 24 h. The content of NO in the culture supernatant was measured using NO assay kit. (A) Effect of 1 nM Prx on NO production. (B) Effects of Prxs at the indicated non-cytotoxic concentrations on NO production. Data are representative of results obtained from three independent experiments with 6 replicates. **P < 0.01 versus control group.

To evaluate the potential inflammatory effect of each of Prxs in macrophages *in vitro*, we first examined the effects of Prx1~Prx6 at the indicated non-cytotoxic concentrations on NO production in RAW264.7 cells. After incubation of 24 h, 1 nM each of Prx showed no obvious effect on NO level (*P* > 0.05; Fig 2A). Moreover, Prx5 and 6 at concentrations of ranging from 5 to 50 nM also had no significant effect on NO production (*P* > 0.05; Fig 2B). However, Prx1, 2 and 4 concentration-dependently induced NO production, resulting in a significant increase in NO levels at a dose of 20 or 50 nM (*P* < 0.01; Fig 2B).

### Proinflammatory cytokine production induced by Prxs in macrophages

**Fig 3:**
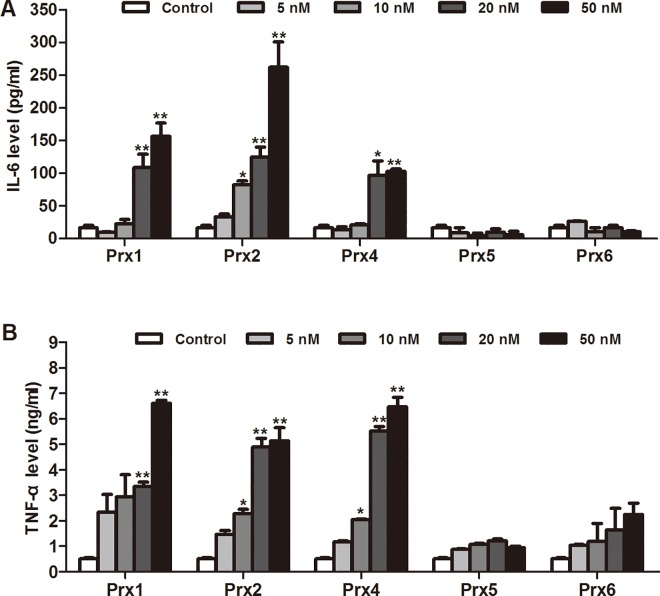
Effects of Prxs on pro-inflammatory cytokine production in murine RAW264.7 macrophages. RAW264.7 cells were treated with Prx at the indicated concentrations for 24 h. The concentrations of pro-inflammatory cytokines in the culture supernatants were determined by ELISA. (A) Effects of Prxs at the indicated non-cytotoxic concentrations on IL-6 production. (B) Effects of Prxs at the indicated non-cytotoxic concentrations on TNF-α production. Data are representative of results obtained from three independent experiments with 6 replicates. *P < 0.05, **P < 0.01 versus control group.

To further explore the proinflammatory activity of each of Prxs in macrophage cells, ELISA was applied to detect the effects of distinct subtype of Prxs (5–50 nM) on proinflammatory cytokine production *in vitro*. Consistent with the induction of NO production by Prx1, 2 and 4, these three subtypes of Prxs also concentration-dependently enhanced the levels of TNF-α and IL-6 in cell culture medium, whereas Prx5 and 6 did not affect cytokine secretion (Fig 3). Treatment with Prx1 (20 or 50 nM), Prx2 (10–50 nM), or Prx4 (10–50 nM) could significantly induce increases in TNF-α and/or IL-6 levels (*P* < 0.05 or *P* < 0.01).

### Effect of LIG on cellular viability in Prx-stimulated macrophages

**Fig 4:**
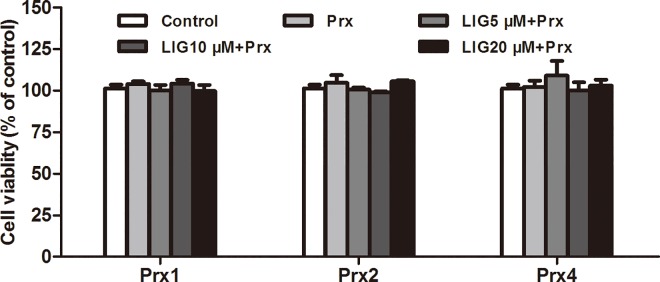
Effect of LIG on cellular viability of murine RAW264.7 macrophages treated with Prxs. RAW264.7 cells were pretreated for 1 h with vehicle or LIG at the indicated concentrations, and then incubated with vehicle (Control) or the indicated Prx (20 nM) for 24 h, respectively. The cell viability was examined by the WST-1 assay and the results are expressed as a percentage of control group. Data are representative of results obtained from three independent experiments with 6 replicates.

To avoid the potential cytotoxic effect of LIG, the effect of LIG on cellular viability was determined in the presence of Prx in RAW264.7 cells. The results showed that LIG at concentrations ranging from 5 to 20 μM had no significant effect on the cellular viability of macrophages co-treated with 20 nM Prx1, Prx2, or Prx4, respectively (*P* > 0.05; Fig 4). These data showed that the results from the corresponding quantitative analysis were not influenced by the cell counts.

### Effect of LIG on proinflammatory mediator production and iNOS expression induced by Prxs in macrophages

**Fig 5:**
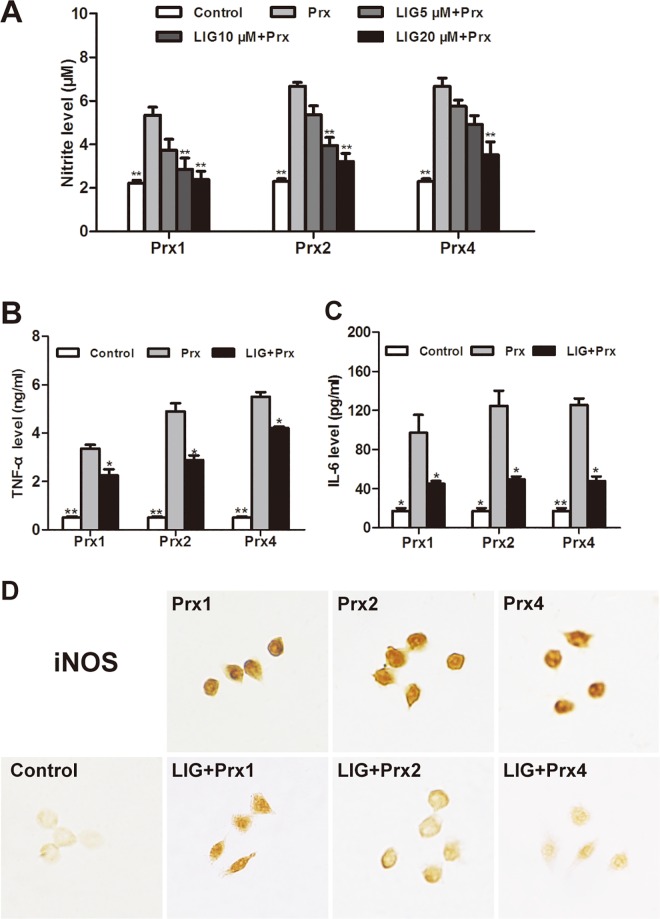
Inhibitory effect of ligustilide (LIG) on Prx-induced proinflammatory mediator production and iNOS expression in murine RAW264.7 macrophages. The cell were pretreated for 1 h with vehicle or LIG, and then incubated with vehicle (Control) or the indicated Prx (20 nM) for 24 h, respectively. (A) Inhibitory effect of LIG (5–20 μM) on Prx-induced NO production in a concentration-dependent manner in the culture medium. (B) & (C) Inhibitory effect of 20 μM LIG on Prx-induced TNF-α and IL-6 production in the culture medium. Data are representative of results obtained from three independent experiments with 6 replicates. *P < 0.05, **P < 0.01 versus the corresponding Prx-treated group. (D) Immunocytochemical photomicrographs of iNOS are representative of 3 independent experiments in RAW264.7 cells.

To study whether LIG could directly inhibit inflammatory response induced by Prx1, 2 and 4 in macrophages *in vitro*, we first investigated the concentration-effect relationship of LIG on NO production in RAW264.7 cells. As shown in Fig 5A, pretreatment of macrophages with LIG at 5, 10, and 20 μM for 1 h prior to Prx (20 nM) stimulation significantly decreased NO production in a concentration-dependent manner compared with the corresponding Prx group, respectively, with maximal inhibitory efficacy at 20 μM LIG (*P* < 0.01). To explore whether proinflammatory cytokines are amenable to modulation by LIG in Prx-stimulated macrophages, the levels of TNF-α and IL-6 in cell culture medium were measured with ELISA. Consistent with the inhibitory effect of LIG on NO production in Prx-stimulated RAW264.7 cells, LIG at a concentration of 20 μM tended to exhibit significant inhibition on the release of proinflammatory cytokines (*P* < 0.05, Fig 5B and 5C).

Moreover, to further confirm the inhibitory effect of LIG on NO production in Prx-treated macrophages, the immunoreactivity of inflammatory enzyme iNOS was detected with immunocytochemistry 24 h after treatment with 20 nM Prx1, 2, or 4, respectively. Fig 5D showed that each of three subtypes of Prxs potently increased iNOS expression, which was significantly inhibited by pretreatment with 20 μM LIG.

### Inhibitory effect of LIG on Prxs-induced TLR4/NF-κB activation in macrophages

**Fig 6:**
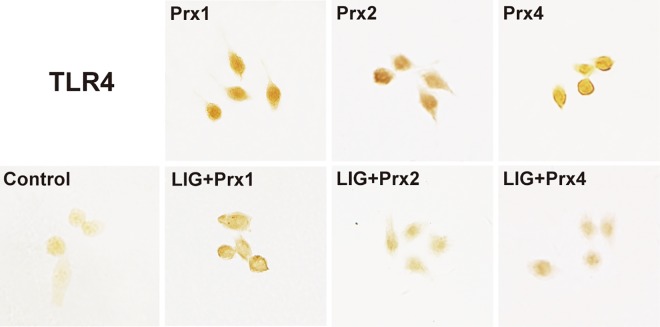
Inhibitory effect of ligustilide (LIG) on Prx-induced TLR4 expression in murine RAW264.7 macrophages. The cells were pretreated for 1 h with vehicle or LIG (20 μM), and then incubated with vehicle (Control) or Prx (20 nM) for 24 h, respectively. TLR4 expression in the cells was detected by immunocytochemistry. Photomicrographs are representative of 3 independent experiments. Pretreatment with 20 μM LIG decreased Prx1, 2 or 4-induced TLR4 expression compared with the corresponding Prx-treated group.

**Fig 7:**
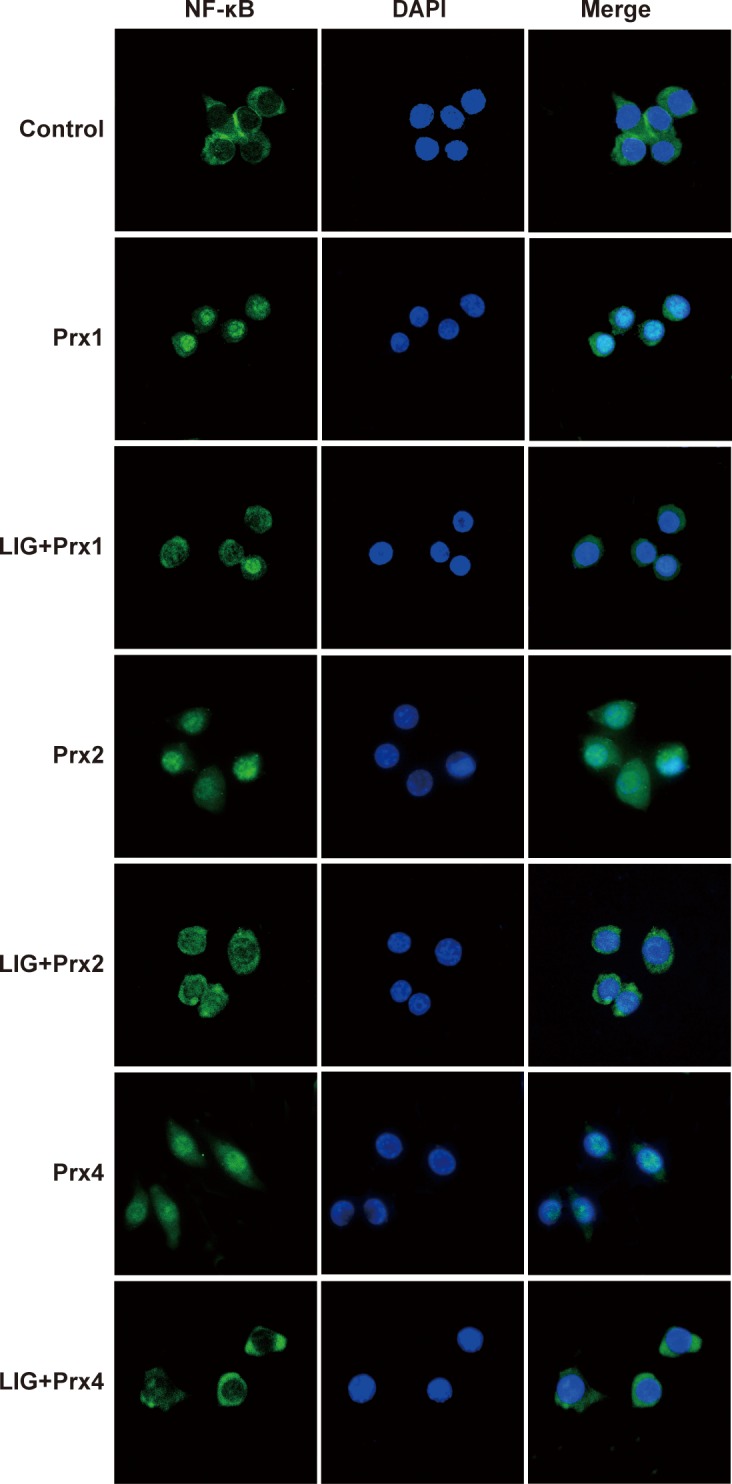
Inhibitory effect of ligustilide (LIG) on Prx-induced NF-κB activation in murine RAW264.7 macrophages. After 1 h exposure to vehicle or 20 μM LIG, RAW264.7 cells were treated with vehicle (Control) or the indicated Prx (20 nM) for 24 h, respectively. Representative photomicrographs of NF-κB subcellular localization visualized using immunofluorescent staining (green) with anti-NF-κB p65 antibody and nuclear DNA staining with DAPI (blue). The images were merged to detect nuclear localization of NF-κB. Pretreatment with 20 μM LIG inhibited Prx1, 2 or 4-induced NF-κB activation compared with the corresponding Prx-treated group.

To investigate the potential mechanism that underlies the anti-inflammatory effect of LIG in Prx-treated macrophages, we further explored the effect of LIG on the TLR4/NF-κB signaling pathway. The TLR4 expression and transcription factor NF-κB activation were examined with immunostaining 24 h after treatment with 20 nM Prx1, 2, or 4, respectively. The present results showed that each of three subtypes of Prxs strongly enhanced the immunoreactivity of TLR4 and nuclear translocation of NF-κB p65 subunit that was predominantly located in the cellular cytoplasm in the control group (Figs 6 and 7). Notably, LIG treatment at a concentration of 20 μM greatly inhibited TLR4 expression and NF-κB activation compared with the corresponding Prx-treated group, suggesting that the inhibitory effect of LIG against Prx-induced inflammation is probably associated with downregulation of TLR4/NF-κB signaling activation in macrophages.

## Discussion

Accumulated evidence suggests that immunomodulation associated with DAMPs/TLRs signaling plays a pivotal role in the destructive neuroinflammatory response accompanying ischemic stroke [6,7,19]. TLR4 is the most widely investigated pattern recognition receptor in ischemic stroke. Studies of TLR4 knockout mice showed smaller infarct sizes and improved neurological deficits after cerebral ischemia [4]. Clinical studies found that TLR4 polymorphisms were associated with ischemic stroke outcome, and TLR4 expression was independently associated with lesion volume and correlated with the intense inflammatory response after ischemic stroke [5]. As a member of highly conserved innate immunity receptors, TLR4 recognizes not only highly preserved structures in infectious pathogens, called pathogen-associated molecular patterns (PAMPs, e.g. LPS), but also DAMPs that are released from aseptic damaged cells. Recent studies have suggested the existence of various types of DAMPs, including nucleic acid, modified lipids, and proteins etc. [19]. Of the known DAMPs in ischemic brain, Prxs have the prominent translational potential at least due to three major reasons: (1) Prxs are highly expressed in brain than in other tissues, which is further upregulated by an increase in oxidative stress after ischemic brain; (2) Prxs are extracellularly released at 12–72 h after stroke onset, which may provide a wide therapeutic window available for the much-needed late phase treatment of ischemic brain injury; (3) the accumulated evidence gradually reveal the potential molecular mechanisms of intracellular and extracellular Prxs [9,19–23].

The previous studies suggest that Prxs, a redox-active intracellular enzyme family, may function as neuroprotective antioxidants within cells in ischemic brain. Several Prxs (Prx2, 3 and 5) have been reported to protect against ischemic cerebral injury through scavenging peroxides and peroxynitrites that is increased by ischemic stress [24–28]. Moreover, intracellular Prx1 could govern the progression of neuroinflammation by suppressing microglial activation in a ROS/p38MAPK-dependent manner, and Prx1 deficiency increased NF-κB mediated-iNOS induction as proinflammatory mediators in microglia [23], suggesting that endogenous Prx1 probably exhibits neuroprotective effect through both anti-oxidative and anti-neuroinflammatory properties. On the other hand, Prxs may act as neurotoxic DAMPs outside cells in ischemic brain. Studies showed extracellular Prxs released from dead or dying brain cells become potent proinflammatory signals to TLR2/4 located predominantly on residential and infiltrating immune cells in ischemic territory, leading to the destructive immune responses [9,14]. Notably, the recent study showed that an increase in Prx2 release could modify the redox status of cell surface receptors and enable induction of inflammatory responses [22]. Collectively, the seemingly paradoxical findings that intracellular and extracellular Prxs have beneficial or harmful effect in progression of ischemic stroke put forward the necessity of further exploring the inflammatory profiles of exogenous Prx subtypes.

Therefore, in the current study, we first measured cell viability by WST-1 assay after treatment with each recombinant mouse Prxs at a range of concentrations from 1 to 50 nM in murine RAW264.7 cells. Strikingly, we observed that Prx3 induced a significant and concentration-dependent cytotoxicity, and 10 nM or more Prx3 caused a great decrease in cellular viability. On the contrary, the rest five members of Prxs at the tested concentrations had no effect on cell viability. Furthermore, we investigated whether each of Prxs at the non-cytotoxic concentrations could induce inflammatory response in macrophages. We found the distinct inflammatory properties of Prxs subtypes in macrophages. The results show that treatment with recombinant Prx3 (1 nM), Prx5 and Prx6 (1 to 50 nM) had no significant effects on the production of proinflammatory mediators (NO, TNF-α or IL-6), iNOS and TLR4 expression, and NF-κB activation, whereas Prx1, 2 and 4 concentration-dependently and significantly induced production of proinflammatory mediators and activation of TLR4/NF-κB signaling in RAW264.7 cells (Figs 2, 3, 5–7 and S2). Other studies have indicated that Prx3, a mitochondrion-specific member of Prxs, plays an indispensable role in apoptotic regulation via direct mitochondrial pathway and interaction with other gene products [29]. The current study shows for the first time that exogenous Prx3 causes the strongest cytotoxicity of Prxs family in macrophages. Further research is needed to explore the cytotoxicity mechanism of Prx3 in future. Altogether, the present results provide the direct and visible evidence to identify the specific members of Prxs with proinflammatory profile in macrophages. Our findings suggest that only Prx1, Prx2 and Prx4 outside cells are potent inflammatory subtypes of Prxs that may directly activate TLR4/NF-κB signaling pathway and trigger inflammatory responses in macrophages.

LPS is a well-characterized PAMP and LPS-stimulated macrophages have widely used as an acute inflammatory model *in vitro* for the evaluation of potential anti-inflammatory agents [15,30]. Similarly, it is reasonable propose that activated macrophages induced by inflammatory DAMPs, such as Prx1, 2 and 4, probably provide a novel cellular model suitable for screening novel anti-inflammatory neuroprotants that inhibit Prxs/TLR4 signaling pathway in ischemic stroke. Natural phthalide compound LIG has been reported to exert potent anti-neuroinflammatory and neuroprotective effects against various cerebral ischemic insults. We and other group have reported that LIG showed a concentration-dependent anti-inflammatory effect in LPS-activated primary microglia and RAW264.7 macrophages *in vitro* [15,30]. An *in vivo* study showed that intravenous LIG injection (20–80 mg/kg) protected rabbits against LPS-induced endotoxic shock, with decreased levels of TNF-α, IL-1β, and nitric oxide and partially restored function of the injured heart, liver, lungs, and kidneys [31]. Moreover, our recent study showed that LIG protected against ischemic brain injury, accompanied by inhibition of the Prx6/TLR4 signaling pathway and neuroinflammatory response [16]. To further investigate whether the neuroprotection of LIG against ischemic brain injury is associated with its direct neutralization on the proinflammatory effect of Prxs, the present study examined the effect of LIG on the inflammatory responses induced by exogenous Prx1, 2, or 4 in RAW264.7 macrophages, respectively. Consistent with the inhibitory effect of LIG against LPS-induced inflammatory response in macrophages [15,30], treatment with LIG (5, 10 or 20 μM) could concentration-dependently and significantly decrease NO release from the macrophages stimulated by Prx1, 2, or 4, respectively. Moreover, 20 μM LIG could significantly inhibited the proinflammatory cytokine production, TLR4 and iNOS expression, and NF-κB activation in the Prx-stimulated cells. Our results suggest that LIG is a small molecule inhibitor targeting TLR4/Prx6 signaling and subsequent inflammatory response, which contributes to its potent protection against neuroinflammation and ischemic brain [16]. The potential effect of LIG on binding of Prx on TLR4 needs to be investigated in future.

Collectively, our study shows that three members of exogenous recombinant Prxs, Prx1, Prx2 and Prx4, are potent inflammatory DAMPs that induce TLR4/NF-κB pathway activation in macrophages, which could be effectively inhibited by LIG treatment. These results suggest that the inflammatory Prx-activated macrophages provide a novel screening model for small molecule inhibitors on aseptic neuroinflammation in ischemic brain. Moreover, the selective blocking strategies targeting the inflammatory Prx/TLR signaling pathway probably provide promising approaches with a wide therapeutic window for ischemic stroke treatment.

## Supporting Information

S1 Fig Chemical structure and high-performance liquid chromatography chromatogram of ligustilide (LIG). The HPLC analysis was performed using a Waters ACQUITY UHPLC system equipped with a photodiode array detector (Milford, MA, USA). LIG was analyzed using a Waters BEH C18 column (1.7 μm, 50 mm × 2.1 mm inner diameter). The isocratic mobile phase consisted of acetonitrile-water (55:45, v/v) at a flow rate of 0.5 ml/min. The column temperature was 30°C, and the detection wavelength was set at 280 nm for acquiring the chromatograms. The purity of LIG was found to be > 98.5%, based on the percentage of total peak area.
(TIF)

S2 Fig Effects of Prx5 and Prx6 on TLR4 and iNOS expression and NF-κB activation in murine RAW264.7 macrophages. The cells were treated with Prx5 or Prx6 (20 nM) for 24 h, respectively, and then harvested for immunostaining. (A) Representative photomicrographs of TLR4 and iNOS expression. (B) Representative photomicrographs of NF-κB subcellular localization visualized using immunofluorescent staining (green) with anti-NF-κB p65 antibody and nuclear DNA staining with DAPI (blue). The images were merged to detect nuclear localization of NF-κB.
(TIF)

## References

[1] Shah IM, Macrae IM, Di Napoli M (2009) Neuroinflammation and neuroprotective strategies in acute ischaemic stroke-from bench to bedside. Curr Mol Med 9: 336–354. 10.2174/156652409787847236 19355915

[2] Lakhan SE, Kirchgessner A, Hofer M (2009) Inflammatory mechanisms in ischemic stroke: therapeutic approaches. J Transl Med 7: 97 10.1186/1479-5876-7-97 19919699 PMC2780998

[3] O'Collins VE, Macleod MR, Donnan GA, Horky LL, van der Worp BH, Howells DW. (2006) 1,026 experimental treatments in acute stroke. Ann Neurol 59: 467–477. 10.1002/ana.20741 16453316

[4] Caso JR, Pradillo JM, Hurtado O, Lorenzo P, Moro MA, Lizasoain I. (2007) Toll-Like Receptor 4 is involved in brain damage and inflammation after experimental stroke. Circulation 115: 1599–1608. 10.1161/CIRCULATIONAHA.106.603431 17372179

[5] Lin YC, Chang YM, Yu JM, Yen JH, Chang JG, Hu CJ (2005) Toll-like receptor 4 gene C119A but not Asp299Gly polymorphism is associated with ischemic stroke among ethnic Chinese in Taiwan. Atherosclerosis 180: 305–309. 10.1016/j.atherosclerosis.2004.12.022 15910856

[6] Eltzschig HK, Eckle T (2011) Ischemia and reperfusion–from mechanism to translation. Nat Med 17: 1391–1401. 10.1038/nm.2507 22064429 PMC3886192

[7] Iadecola C, Anrather J (2011) The immunology of stroke: from mechanisms to translation. Nat Med 17: 796–808. 10.1038/nm.2399 21738161 PMC3137275

[8] Qiu J, Nishimura M, Wang Y, Sims JR, Qiu S, Savitz SI, et al (2008) Early release of HMGB-1 from neurons after the onset of brain ischemia. J Cereb Blood Flow Metab 28: 927–938. 10.1038/sj.jcbfm.9600582 18000511

[9] Shichita T, Hasegawa E, Kimura A, Morita R, Sakaguchi R, Takada I, et al (2012) Peroxiredoxin family proteins are key initiators of post-ischemic inflammation in the brain. Nat Med 18: 911–917. 10.1038/nm.2749 22610280

[10] Wood ZA, Schröder E, Robin Harris J, Poole LB (2003) Structure, mechanism and regulation of peroxiredoxins. Trends Biochem Sci 28: 32–40. 10.1016/s0968-0004(02)00003-8 12517450

[11] Sarafian TA, Verity MA, Vinters HV, Shih CC, Shi L, Ji XD, et al (1999) Differential expression of peroxiredoxin subtypes in human brain cell types. J Neurosci Res 56: 206–212. 10.1002/(sici)1097-4547(19990415)56:2<206::aid-jnr10>3.0.co;2-x 10494109

[12] Jin MH, Lee YH, Kim JM, Sun HN, Moon EY, Shong MH, et al (2005) Characterization of neural cell types expressing peroxiredoxins in mouse brain. Neurosci Lett 381: 252–257. 10.1016/j.neulet.2005.02.048 15896479

[13] Lin M, Zhu GD, Sun QM, Fang QC (1979) Chemical studies of angelica sinensis. Acta Pharmceut Sin 14: 529–534. 532655

[14] Kuang X, Du JR, Liu YX, Zhang GY, Peng HY (2008) Postischemic administration of Z-Ligustilide ameliorates cognitive dysfunction and brain damage induced by permanent forebrain ischemia in rats. Pharmacol Biochem Behav 88: 213–221. 10.1016/j.pbb.2007.08.006 17889286

[15] Wang J, Du JR, Wang Y (2010) Z-ligustilide attenuates lipopolysaccharide- induced proinflammatory response via inhibiting NF-kappaB pathway in primary rat microglia. Acta Pharmacol Sin 31: 791–797. 10.1038/aps.2010.71 20581853 PMC4007734

[16] Kuang X, Wang LF, Yu L, Li YJ, Wang YN, He Q, et al (2014) Ligustilide ameliorates neuroinflammation and brain injury in focal cerebral ischemia/reperfusion rats: involvement of inhibition of TLR4/peroxiredoxin 6 signaling. Free Radic Biol Med 71: 165–175. 10.1016/j.freeradbiomed.2014.03.028 24681253

[17] Miyamoto T, Kashima H, Yamada Y, Kobara H, Asaka R, Ando H, et al (2016) Lipocalin 2 enhances migration and resistance against cisplatin in endometrial carcinoma cells. PLOS ONE 11:e0155220 10.1371/journal.pone.0155220 27168162 PMC4864227

[18] Kuang X, Chen YS, Wang LF, Li YJ, Liu K, Zhang MX, et al (2014) Klotho upregulation contributes to the neuroprotection of ligustilide in an Alzheimer's disease mouse model. Neurobiol Aging 35:169–178. 10.1016/j.neurobiolaging.2013.07.019 23973442

[19] Shichita T, Ito M, Yoshimura A (2014) Post-ischemic inflammation regulates neural damage and protection. Front Cell Neurosci 8: 319 10.3389/fncel.2014.00319 25352781 PMC4196547

[20] Riddell JR, Wang XY, Minderman H, Gollnick SO (2010) Peroxiredoxin 1 stimulates secretion of proinflammatory cytokines by binding to TLR4. J Immunol 184: 1022–1030. 10.4049/jimmunol.0901945 20018613 PMC2955897

[21] Garcia-Bonilla L, Iadecola C (2012) Peroxiredoxin sets the brain on fire after stroke. Nat Med 18: 858–859. 10.1038/nm.2797 22673994 PMC3955950

[22] Salzano S, Checconi P, Hanschmann EM, Lillig CH, Bowler LD, Chan P, et al (2014) Linkage of inflammation and oxidative stress via release of glutathionylated peroxiredoxin-2, which acts as a danger signal. Proc Natl Acad Sci USA 111: 12157–12162. 10.1073/pnas.1401712111 25097261 PMC4143057

[23] Kim SU, Park YH, Min JS, Sun HN, Han YH, Hua JM, et al (2013) Peroxiredoxin I is a ROS/p38 MAPK-dependent inducible antioxidant that regulates NF-kappaB-mediated iNOS induction and microglial activation. J Neuroimmunol 259: 26–36. 10.1016/j.jneuroim.2013.03.006 23602274

[24] Bryk R, Griffin P, Nathan C (2000) Peroxynitrite reductase activity of bacterial peroxiredoxins. Nature 407: 211–215. 10.1038/35025109 11001062

[25] Winterbourn C.C. (2008) Reconciling the chemistry and biology of reactive oxygen species. Nat Chem Biol 4: 278–286. 10.1038/nchembio.85 18421291

[26] Gan Y, Ji X, Hu X, Luo Y, Zhang L, Li P, et al (2012) Transgenic overexpression of peroxiredoxin-2 attenuates ischemic neuronal injury via suppression of a redox-sensitive pro-death signaling pathway. Antioxid Redox Signal 17: 719–732. 10.1089/ars.2011.4298 22356734 PMC3387778

[27] Hattori F, Murayama N, Noshita T, Oikawa S (2003) Mitochondrial peroxiredoxin-3 protects hippocampal neurons from excitotoxic injury in vivo. J Neurochem 86: 860–868. 10.1046/j.1471-4159.2003.01918.x 12887684

[28] Plaisant F, Clippe A, Van der Stricht D, Knoops B, Gressens P (2003) Recombinant peroxiredoxin 5 protects against excitotoxic brain lesions in newborn mice. Free Radic Biol Med 3: 862–872. 10.1016/s0891-5849(02)01440-5 12654475

[29] Noh YH, Baek JY, Jeong W, Rhee SG, Chang TS (2009) Sulfiredoxin translocation into mitochondria plays a crucial role in reducing hyperoxidized peroxiredoxin III. J Biol Chem 284:8470–8477. 10.1074/jbc.M808981200 19176523 PMC2659205

[30] Su YW, Chiou WF, Chao SH, Lee MH, Chen CC, Tsai YC. (2011) Ligustilide prevents LPS-induced iNOS expression in RAW 264.7 macrophages by preventing ROS production and down-regulating the MAPK, NF-κB and AP-1 signaling pathways. Int Immunopharmacol 11: 1166–1172. 10.1016/j.intimp.2011.03.014 21457761

[31] Shao M, Qu K, Liu K, Zhang Y, Zhang L (2011) Effects of ligustilide on lipopolysaccharide-induced endotoxic shock in rabbits. Planta Med 77: 809–816. 10.1055/s-0030-1250573 21104607

