# Supplementary figures and images for: Differences in Proinflammatory Property of Six Subtypes of Peroxiredoxins and Anti-Inflammatory Effect of Ligustilide in Macrophages
Source: PLoS One. 2016 Oct 7;11(10):e0164586. doi: 10.1371/journal.pone.0164586 (PMC5055302; doi:10.1371/journal.pone.0164586)

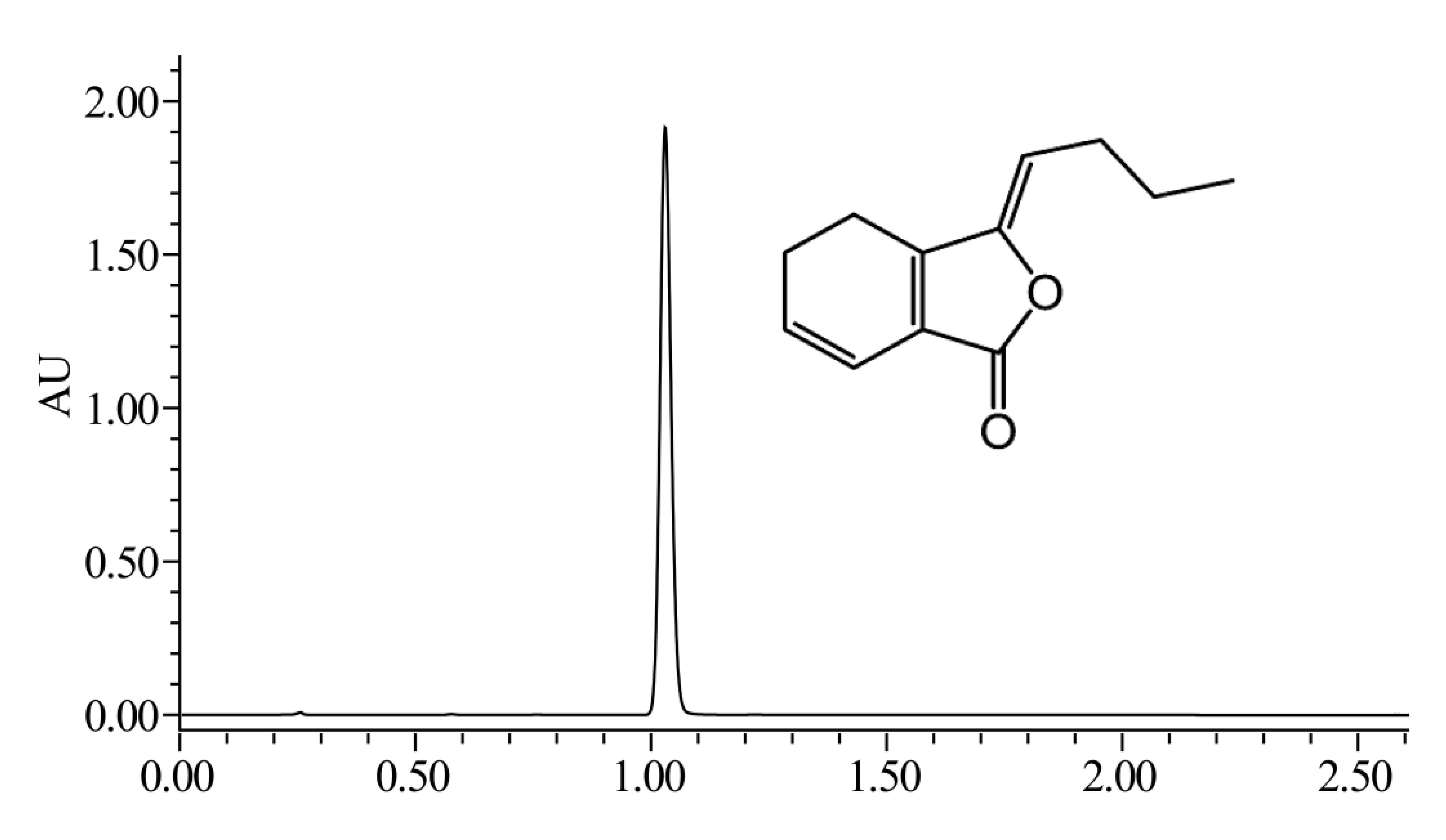

Supplement: S1 Fig — The HPLC analysis was performed using a Waters ACQUITY UHPLC system equipped with a photodiode array detector (Milford, MA, USA). LIG was analyzed using a Waters BEH C18 column (1.7 μm, 50 mm × 2.1 mm inner diameter). The isocratic mobile phase consisted of acetonitrile-water (55:45, v/v) at a flow rate of 0.5 ml/min. The column temperature was 30°C, and the detection wavelength was set at 280 nm for acquiring the chromatograms. The purity of LIG was found to be > 98.5%, based on the percentage of total peak area. (TIF) [file pone.0164586.s001.tif]

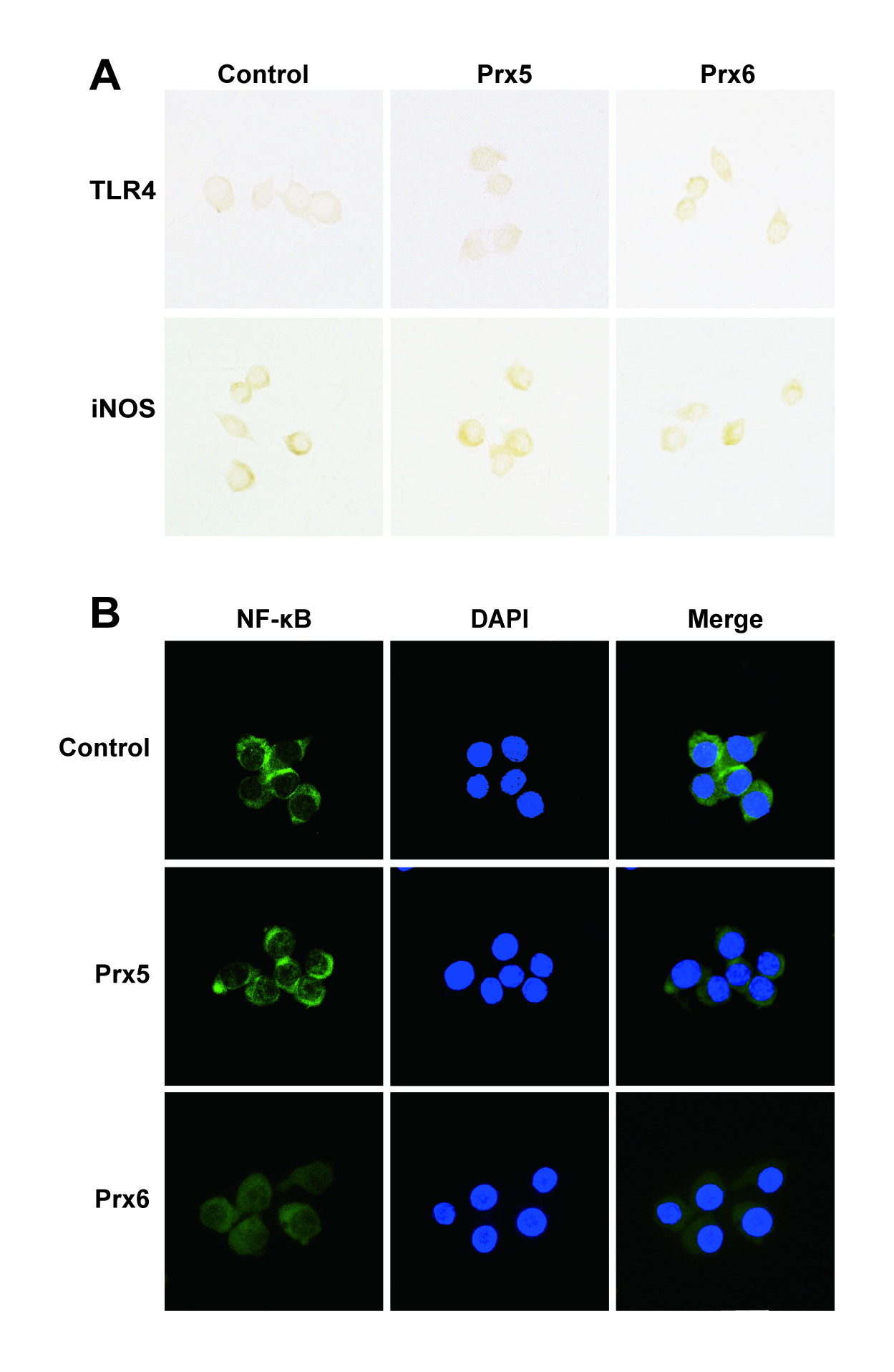

Supplement: S2 Fig — The cells were treated with Prx5 or Prx6 (20 nM) for 24 h, respectively, and then harvested for immunostaining. (A) Representative photomicrographs of TLR4 and iNOS expression. (B) Representative photomicrographs of NF-κB subcellular localization visualized using immunofluorescent staining (green) with anti-NF-κB p65 antibody and nuclear DNA staining with DAPI (blue). The images were merged to detect nuclear localization of NF-κB. (TIF) [file pone.0164586.s002.tif]
